# Analysis and Allocation of Cancer-Related Genes Using Vague DNA Sequence Data

**DOI:** 10.3389/fgene.2022.858005

**Published:** 2022-04-19

**Authors:** Muhammad Aslam, Mohammed Albassam

**Affiliations:** Department of Statistics, Faculty of Science, King Abdulaziz University, Jeddah, Saudi Arabia

**Keywords:** multinomial distribution, chi-square test, classical statistics, neutrosophy, DNA data

## Abstract

To test the equality of several independent multinomial distributions, the chi-square test for count data is applied. The existing test can be applied when complete information about the data is available. The complex process, such as DNA count, the existing test under classical statistics may mislead. To overcome the issue, the modification of the chi-square test for multinomial distribution under neutrosophic statistics is presented in this paper. The modified form of the chi-square test statistic under indeterminacy/uncertainty is presented and applied using the DNA count data. From the DNA count data analysis, simulation, and comparative studies, the proposed test is found to be informative, springy, and good as compared with the existing tests.

## Introduction

Without statistical analysis, it is not possible to check the significance of variables under study. For testing the significance of variables, statistical tests are applied in a variety of fields (Ali & Bhaskar, 2016 and Greenland et al., 2016). The chi-square test for multinomial distribution is applied for testing whether the allocation of objects to different groups is equally likely or not. This test is applied for testing the null hypothesis that allocation of objects to different groups is equal vs. the alternative hypothesis that allocation of objects to different groups is unequal. The test statistic is computed from the data, and the null hypothesis is accepted if the values of the statistic fall within the acceptance region. Cohen, Kolassa, & Sackrowitz (2006) use the test for equality of multinomial distributions. Chafai & Concordet (2009) study confidence intervals for multinomial distribution in the case of small samples. Turner, Deng, & Houle (2020) use the statistical tests for head and face data. Shin, Yamamoto, Brady, Lee, & Haynes (2019) and Mollan et al. (2019) discuss the applications of statistical tests.

Statistical methods are widely used in analyzing and testing the significance of DNA data. A rich literature of statistical methods analyzing DNA data is available. Goldman (1993a) applies statistical tests using DNA data. Buldyrev et al. (1998) and Kugiumtzis & Provata (2004) analyze DNA data using statistical physics. Yoshida, Kobayashi, Futagami, & Fujikoshi (1999) use statistical analysis for DNA data. Pai, Mathew, & Anindya (2021) work on prediction using DNA data. Yao, Jin, & Lee (2018) improve the statistical analysis for genetic data. Gunasekaran et al. (2021) analyze DNA data using hybrid models. Halla-aho and Lähdesmäki (2021) use statistical analysis for DNA cancer data. More applications of the statistical techniques for DNA data can be seen in Goldman (1993b), Keinduangjun, Piamsa-nga, & Poovorawan (2005), Rodriguez et al. (2012), and Pai et al. (2021).

Fuzzy-based statistical tests are applied when the data in hand has vague or incomplete information. Viertl (2006) mentions that “statistical data are frequently not precise numbers but more or less non-precise also called fuzzy. Measurements of continuous variables are always fuzzy to a certain degree.” Several studies using fuzzy-based multinomial distribution are available in the literature. Amirzadeh, Mashinchi, & Yaghoobi (2008) study multinomial distribution using fuzzy logic. Mashuri & Ahsan (2018) work on a fuzz-based chart using multinomial distribution. More information for fuzzy-based multinomial distribution can be seen in Amirzadeh et al. (2008) and Hrafnkelsson, Oddsson, & Unnthorsson (2016).

Smarandache (2013) discusses that neutrosophic logic is more efficient than interval- and fuzzy-based analysis. Neutrosophic statistics are applied to analyze the data having neutrosophic numbers; see F Smarandache (2014). Interval statistics use interval data to capture the data in the interval only and are silent about the measure of indeterminacy. On the other hand, fuzzy-based analysis only gives information about the measure of truth and of falseness. Neutrosophic statistics become classical statistics when no indeterminate information is found in the data. Chen et al. (2017a,b) introduced the methods to deal with the neutrosophic data. Later on, Sherwani et al. (2021), Aslam (2021), and Albassam, Khan, & Aslam (2021) introduced statistical tests under neutrosophic statistics.

The chi-square test for multinomial distribution available in the literature can be applied when full information about data is given. Complex processes or processes under uncertainty do not possess the full information about the data or level of significance. Therefore, there is a gap in the design of the chi-square test for multinomial distribution under neutrosophic statistics. Therefore, in this study, the chi-square test for multinomial distribution using neutrosophic statistics is introduced the first time according to the best of the author’s knowledge. The application of the proposed test is given with the aid of DNA cancer data. It is expected the proposed test will be more competent than the existing tests in terms of springy, deftness, and goodness.

## Methods

The existing test for the equality of multinomial distribution can only be utilized when no vague information is presented. To overcome this issue, modification of the existing test is necessary. In this section, modification of the existing test under classical statistics is presented under neutrosophic statistics. With the expectation that the proposed test for the equality of multinomial distribution performs better for testing the null hypothesis under an uncertain environment. The main objective of the paper is to introduce the test for the equality of $h_{N}$ independent neutrosophic multinomial distributions. Let $Y_{1jN},Y_{2jN}\ldots Y_{kjN}\left(j=1,2,\ldots ,h_{N}\right)$ present the neutrosophic frequencies for the neutrosophic events $A_{1N},A_{2N}\ldots A_{kN}$ . Let $p_{ijN}=P\left(A_{iN}\right)$ ; $i_{N}=1,2,\ldots ,k_{N};j_{N}=1,2,\ldots ,h_{N}$ . The neutrosophic form of $p_{ijN}\varepsilon \left[p_{ijL},p_{ijU}\right]$ is expressed as

$$p_{ijN}=p_{ijL}+p_{ijU}I_{p_{ijN}};I_{p_{ijN}}\varepsilon \left[I_{p_{ijL}},I_{p_{ijU}}\right]$$ (1)

where $p_{ijL}$ presents the determined part, and $p_{ijU}I_{p_{ijN}}$ presents the indeterminate part and $I_{p_{ijN}}\varepsilon \left[I_{p_{ijL}},I_{p_{ijU}}\right]$ is the measure of indeterminacy. The alternative expression of Eq. 1 can be given as

$$p_{ijN}=\left(1+I_{p_{ijN}}\right)p_{ij};I_{p_{ijN}}\varepsilon \left[I_{p_{ijL}},I_{p_{ijU}}\right]$$ (2)

The $j_{th}$ experiment is carried out $n_{jN}$ times under the assumption that $n_{jN}$ instances are independent. The modified form of the test statistic $Q_{N}\varepsilon \left[Q_{L},Q_{U}\right]$ is expressed as follows:

$$Q_{N}=Q_{L}+Q_{U}I_{Q_{N}};I_{Q_{N}}\varepsilon \left[I_{Q_{L}},I_{Q_{U}}\right]$$ (3)

where

$$Q_{N}=\overset{h_{N}}{\underset{j=1}{\sum }}\overset{k_{N}}{\underset{i=1}{\sum }}\frac{{\left(Y_{ijN}-n_{jN}p_{ijN}\right)}^{2}}{n_{jN}p_{ijN}}$$

The proposed statistic $Q_{N}\varepsilon \left[Q_{L},Q_{U}\right]$ can be written as

$$Q_{N}=\overset{h_{L}}{\underset{j=1}{\sum }}\overset{k_{L}}{\underset{i=1}{\sum }}\frac{{\left(Y_{ijL}-n_{jL}p_{ijL}\right)}^{2}}{n_{jL}p_{ijL}}+\overset{h_{U}}{\underset{j=1}{\sum }}\overset{k_{U}}{\underset{i=1}{\sum }}\frac{{\left(Y_{ijU}-n_{jU}p_{ijU}\right)}^{2}}{n_{jU}p_{ijU}}I_{Q_{N}};I_{Q_{N}}\varepsilon \left[I_{Q_{L}},I_{Q_{U}}\right]$$ (4)

The simplified form of statistic can be written as

$$Q_{N}=\left(1+I_{Q_{N}}\right)\overset{h_{N}}{\underset{j=1}{\sum }}\overset{k_{N}}{\underset{i=1}{\sum }}\frac{{\left(Y_{ijN}-n_{jN}p_{ijN}\right)}^{2}}{n_{jN}p_{ijN}};I_{Q_{N}}\varepsilon \left[I_{Q_{L}},I_{Q_{U}}\right]$$ (5)

Note that the proposed test $Q_{N}\varepsilon \left[Q_{L},Q_{U}\right]$ is a generalization of the test under classic statistics. The proposed test $Q_{N}\varepsilon \left[Q_{L},Q_{U}\right]$ reduces to the classic test under classic statistics when $I_{Q_{L}}$ = 0. The proposed test is also a generalization of the tests under interval statistics and fuzzy-based logic. The proposed test $Q_{N}\varepsilon \left[Q_{L},Q_{U}\right]$ follows the neutrosophic chi-square distribution with $h_{N}\left(k_{N}-1\right)$ degree of freedom. The proposed test $Q_{N}\varepsilon \left[Q_{L},Q_{U}\right]$ is applied to test the following null hypothesis:

$$H_{0N}:p_{i1}=p_{i2}=\ldots =p_{ih_{N}}=p_{iN},\;\;\;i=1,2,3,\ldots ,k_{N}$$ (6)

Under the null hypothesis, we estimate $k_{N}-1$ probabilities from

$${\hat{p}}_{iN}=\frac{\sum_{j=1}^{h_{L}}Y_{ijL}}{\sum_{j=1}^{h_{L}}n_{jL}}+\frac{\sum_{j=1}^{h_{U}}Y_{ijU}}{\sum_{j=1}^{h_{U}}n_{jU}}I_{{\hat{p}}_{iN}};I_{{\hat{p}}_{iN}}\varepsilon \left[I_{{\hat{p}}_{iL}},I_{{\hat{p}}_{iU}}\right]$$ (7)

The statistic $Q_{N}\varepsilon \left[Q_{L},Q_{U}\right]$ based on ${\hat{p}}_{iN}\varepsilon \left[{\hat{p}}_{iL},{\hat{p}}_{iU}\right]$ is expressed as

$$Q_{N}=\overset{h_{L}}{\underset{j=1}{\sum }}\overset{k_{L}}{\underset{i=1}{\sum }}\frac{{\left(Y_{ijL}-n_{jL}{\hat{p}}_{ijL}\right)}^{2}}{n_{jL}{\hat{p}}_{ijL}}+\overset{h_{U}}{\underset{j=1}{\sum }}\overset{k_{U}}{\underset{i=1}{\sum }}\frac{{\left(Y_{ijU}-n_{jU}{\hat{p}}_{ijU}\right)}^{2}}{n_{jU}{\hat{p}}_{ijU}}I_{Q_{N}};I_{Q_{N}}\varepsilon \left[I_{Q_{L}},I_{Q_{U}}\right]$$ (8)

The simplified form of statistic can be written as

$$Q_{N}=\left(1+I_{Q_{N}}\right)\overset{h_{N}}{\underset{j=1}{\sum }}\overset{k_{N}}{\underset{i=1}{\sum }}\frac{{\left(Y_{ijN}-n_{jN}{\hat{p}}_{ijN}\right)}^{2}}{n_{jN}{\hat{p}}_{ijN}};I_{Q_{N}}\varepsilon \left[I_{Q_{L}},I_{Q_{U}}\right]$$ (9)

Note that $Q_{N}\varepsilon \left[Q_{L},Q_{U}\right]$ based on ${\hat{p}}_{iN}\varepsilon \left[{\hat{p}}_{iL},{\hat{p}}_{iU}\right]$ follows the neutrosophic chi-square distribution with $\left(h_{N}-1\right)\left(k_{N}-1\right)$ degree of freedom.

## Application

In this section, the application of the proposed test is given using DNA sequence data. The data is related to the cancer-related gene BRCA 2. According to https://medlineplus.gov/genetics/gene/brca2/#:∼:text=Mutations%20in%20the%20BRCA2%20gene,one%20generation%20to%20the%20next “Mutations in the BRCA2 gene are associated with an increased risk of breast cancer in both men and women, as well as several other types of cancer. These mutations are present in every cell in the body and can be passed from one generation to the next.” By following https://www.math.mcgill.ca/∼dstephens/OldCourses/204-2007/Handouts/Math204-ChiSquareWithResults.pdf, the counts of nucleotide (A, C, G, T) having two counting groups are reported in Table 1. Note here that, in Table 1, the data given in “Count Group 1” is selected from the given reference, and the data given in “Count Group 2” is generated by simulation. The DNA sequence is a complex process, and there may be uncertainty/indeterminacy in counts; see Yurov, Vorsanova, & Iourov (2011). In the presence of uncertainty/indeterminacy in counts, the proposed test can be applied more effectively than the existing test under classic statistics. Suppose that there is 5% uncertainty/indeterminacy in counts of the numbers of nucleotides (A, C, G, T) in the DNA sequence of the cancer-related gene BRCA 2. Based on the information and data given in Table 1, the proposed test statistic is calculated as follows:

$$\overset{4}{\underset{j=1}{\sum }}\overset{4}{\underset{i=1}{\sum }}\frac{{\left(Y_{ijL}-n_{jL}{\hat{p}}_{ijL}\right)}^{2}}{n_{jL}{\hat{p}}_{ijL}}=\text{0}\text{.}000365921+\text{0}\text{.}002051303+\ldots +\text{0}\text{.}000748132=\text{0}\text{.}00664$$

**TABLE 1:** The counts of nucleotide data.

Category	1	2	3	4	Total
Nucleotide	A	C	G	T	
Count Group 1	38,514	24,631	25,685	38,249	127,079
Count Group 2	38,550	24,635	25,700	38,288	127,173

The statistic $Q_{N}\varepsilon \left[Q_{L},Q_{U}\right]$ in neutrosophic form can be expressed as follows:

$$Q_{N}=0.00664+0.00664I_{Q_{N}};I_{Q_{N}}\varepsilon \left[0,0.05\right]$$

The simplified form of statistic can be written as

$$Q_{N}=\left(1+0.05\right)0.00664=0.00697;I_{Q_{N}}\varepsilon \left[0,0.05\right]$$

The proposed test DNA count data is implemented in the following steps.

**Step 1:** State the null hypothesis $H_{0}$ : The allocation of DNA count is equally likely vs. the alternative hypothesis $H_{1}:\;$ The allocation of DNA count is unequal.

**Step 2:** The level of significance α = 0.05 and the tabulated value from Kanji (2006) is 9.35.

**Step 3:** Compute the value of statistic $Q_{N}$ = 0.00697 and compare it with the tabulated value.

**Step 4:** As the computed value of $Q_{N}$ is less than 9.35, $H_{0}$ is accepted.

Based on the analysis, it can be concluded that there is no evidence to suspect unequal allocation of counts of nucleotide (A, C, G, T).

## Simulation Study

A simulation study is performed to assess the effect of indeterminacy $I_{Q_{N}}$ in counts of the numbers of nucleotides (A, C, G, T) in the DNA sequence of the cancer-related gene BRCA 2 on the statistic $Q_{N}$ . To see the effect of $I_{Q_{N}}$ on the statistic $Q_{N}$ , various values of $I_{Q_{N}}$ are considered. Using the neutrosophic form obtained for the DNA count data, the values of statistic $Q_{N}$ are shown in Table 2. From Table 2, it can be noted that, as the value indeterminacy $I_{Q_{N}}$ increases, the values of $Q_{N}$ also increase. The decision about $H_{0}$ at various values of $I_{Q_{N}}$ is also shown in Table 2. From Table 2, although the values of statistic $Q_{N}$ increase as $I_{Q_{N}}$ increases, but it does not change the decision about the acceptance $H_{0}$ .

**TABLE 2:** The effect of Indeterminacy on $Q_{N}$ .

$I_{Q_{N}}$	$Q_{N}$	Decision about $H_{0}$	$I_{Q_{N}}$	$Q_{N}$	Decision about $H_{0}$
(0, 0)	(0.00664, 0.00664)	Do not reject $H_{0}$	(0, 0.1)	(0.00664, 0.007304)	Do not reject $H_{0}$
(0, 0.01)	(0.00664, 0.006706)	Do not reject $H_{0}$	(0, 0.2)	(0.00664, 0.007968)	Do not reject $H_{0}$
(0, 0.02)	(0.00664, 0.006773)	Do not reject $H_{0}$	(0, 0.3)	(0.00664, 0.008632)	Do not reject $H_{0}$
(0, 0.03)	(0.00664, 0.006839)	Do not reject $H_{0}$	(0, 0.4)	(0.00664, 0.009296)	Do not reject $H_{0}$
(0, 0.04)	(0.00664, 0.006906)	Do not reject $H_{0}$	(0, 0.5)	(0.00664, 0.00996)	Do not reject $H_{0}$
(0, 0.05)	(0.00664, 0.006972)	Do not reject $H_{0}$	(0, 0.6)	(0.00664, 0.010624)	Do not reject $H_{0}$
(0, 0.06)	(0.00664, 0.007038)	Do not reject $H_{0}$	(0, 0.7)	(0.00664, 0.011288)	Do not reject $H_{0}$
(0, 0.07)	(0.00664, 0.007105)	Do not reject $H_{0}$	(0, 0.8)	(0.00664, 0.011952)	Do not reject $H_{0}$
(0, 0.08)	(0.00664, 0.007171)	Do not reject $H_{0}$	(0, 0.9)	(0.00664, 0.012616)	Do not reject $H_{0}$
(0, 0.09)	(0.00664, 0.007238)	Do not reject $H_{0}$	(0, 1)	(0.00664, 0.01328)	Do not reject $H_{0}$

## Comparative Studies

The springy, deftness, and goodness of the proposed test over the tests under interval statistics, the fuzzy-based approach, and classic statistics is shown in this section. The efficiency of the proposed test is shown in terms of the measure of indeterminacy, springyness, deftness, and goodness. The neutrosophic form of the statistic $Q_{N}\varepsilon \left[Q_{L},Q_{U}\right]$ is expressed as follows:

$$Q_{N}=0.00664+0.00664I_{Q_{N}};I_{Q_{N}}\varepsilon \left[0,0.05\right]$$

The abovementioned neutrosophic form is based on two types of information. The first part, 0.00664 , gives information about the determinate part, and the second part, $0.00664I_{Q_{N}}$ , gives information about the indeterminate part. The proposed statistic $Q_{N}\varepsilon \left[Q_{L},Q_{U}\right]$ reduces to the test under classic statistics when $I_{Q_{L}}$ = 0. Therefore, it can be analyzed that the existing test under classic statistics gives only information about the determinate part. On the other hand, the proposed test gives information about the indeterminacy additionally as compared with the test using classic statistics. Therefore, the proposed test is more bendable than the existing test under classic statistics. The interval statistics only utilize the information given in the interval. In simple words, the interval statistics capture the information between intervals. Now comparing the results of the proposed test under the test statistic under interval statistics, it can be seen that the proposed test is more explanatory than the test using interval statistics as earlier it did not give any information about the measure of indeterminacy. Therefore, the proposed test is also more efficient than the test using the interval-based statistic. The test statistic using fuzzy logic can be considered measures of truth and falseness. The neutrosophic statistics use the set analysis and can be used for any type of set. The proposed statistic $Q_{N}\varepsilon \left[Q_{L},Q_{U}\right]$ gives three types of information. The proposed test states that the chance of accepting $H_{0}$ is 0.95 (a measure of truth), the chance of committing a type-I error is 0.05 (a measure of falseness), and the measure of indeterminacy associated with the test is 0.05. From the study, it is concluded that the proposed test is also a generalization of the test using fuzzy logic. Therefore, the proposed test is more informative than the three existing tests.

## Concluding Remarks

The modification of the existing test for the equality of multinomial distribution under neutrosophic statistics is introduced in the paper. The proposed test is the generalization of several existing tests under interval statistics, fuzzy-based, and classic statistics. The modification of the test statistic is presented in the presence of indeterminacy. The simulation and comparative studies show that the proposed test is adequate and effective to apply in the presence of uncertainty. The application of the proposed test for DNA count data also shows its efficiency. The proposed test can be applied for testing the allocation of count is equally likely or not in medical science, engineering, and political science. More properties of the proposed test can be studied in future research. The proposed test using a double sampling scheme is another fruitful area for future research.

## Data Availability

The original contributions presented in the study are included in the article/Supplementary Material, further inquiries can be directed to the corresponding author.
